# Pyramidal neurons proportionately alter the identity and survival of specific cortical interneuron subtypes

**DOI:** 10.21203/rs.3.rs-4774421/v1

**Published:** 2024-08-02

**Authors:** Sherry Jingjing Wu, Min Dai, Shang-Po Yang, Cai McCann, Yanjie Qiu, Giovanni J. Marrero, Jeffrey A. Stogsdill, Daniela J. Di Bella, Qing Xu, Samouil L. Farhi, Evan Z. Macosko, Fei Che, Gord Fishell

**Affiliations:** 1 Harvard Medical School, Blavatnik Institute, Department of Neurobiology, Boston, MA 02115, USA.; 2 Stanley Center for Psychiatric Research, Broad Institute of MIT and Harvard, Cambridge, MA 02142, USA.; 3 Spatial Technology Platform, Broad Institute of MIT and Harvard, Cambridge, MA 02142, USA.; 4 Broad Institute of MIT and Harvard, Cambridge, MA 02142, USA; 5 Center for Genomics & Systems Biology, New York University Abu Dhabi, Abu Dhabi, UAE; 6 Department of Stem Cell and Regenerative Biology, Harvard University, Cambridge, MA 02138, USA

## Abstract

The mammalian cerebral cortex comprises a complex neuronal network that maintains a delicate balance between excitatory neurons and inhibitory interneurons. Previous studies, including our own research, have shown that specific interneuron subtypes are closely associated with particular pyramidal neuron types, forming stereotyped local inhibitory microcircuits. However, the developmental processes that establish these precise networks are not well understood. Here we show that pyramidal neuron types are instrumental in driving the terminal differentiation and maintaining the survival of specific associated interneuron subtypes. In a wild-type cortex, the relative abundance of different interneuron subtypes aligns precisely with the pyramidal neuron types to which they synaptically target. In *Fezf2* mutant cortex, characterized by the absence of layer 5 pyramidal tract neurons and an expansion of layer 6 intratelencephalic neurons, we observed a corresponding decrease in associated layer 5b interneurons and an increase in layer 6 subtypes. Interestingly, these shifts in composition are achieved through mechanisms specific to different interneuron types. While SST interneurons adjust their abundance to the change in pyramidal neuron prevalence through the regulation of programmed cell death, parvalbumin interneurons alter their identity. These findings illustrate two key strategies by which the dynamic interplay between pyramidal neurons and interneurons allows local microcircuits to be sculpted precisely. These insights underscore the precise roles of extrinsic signals from pyramidal cells in the establishment of interneuron diversity and their subsequent integration into local cortical microcircuits.

Single-cell sequencing studies have elucidated the transcriptomic landscape of cortical neurons across the isocortex, revealing considerable diversity among both excitatory neurons and inhibitory interneurons^1–3^. These studies also showed that while excitatory neuron subtypes exhibit clear transcriptomic variations across different cortical regions, interneuron subtypes maintain a consistent profile independent of the cortical region examined^3^. Nonetheless, previous studies demonstrated that interneurons form subtype-specific synaptic connections with particular excitatory neuron populations, a preference observed across various regions of the cortex^4–10^. The ability of clonally-related interneurons to adopt different identities and integrate precisely with distinct excitatory neurons present in specific cortical regions^11–15^ implies a crucial role for excitatory neurons in directing the development of interneuron subtypes. Our quantitative analysis of different interneuron subtypes showed that interneuron subtypes in different cortical regions generally adapt to the composition of local pyramidal neurons (PNs). To directly test the influence of PNs on interneuron diversity, we employed genetic strategies to selectively alter the identity of specific PN types and demonstrated that the quantity of particular PVALB and SST interneuron subtypes changed in accordance with this shift. Importantly, these changes occur through distinct mechanisms: PNs promote the survival of their partner SST interneuron subtypes, while inducing fate changes in PVALB interneuron populations towards their preferred subtypes.

## Interneuron subtypes exhibit consistent laminar distributions across cortical regions

**Extended Data Fig. 1:**
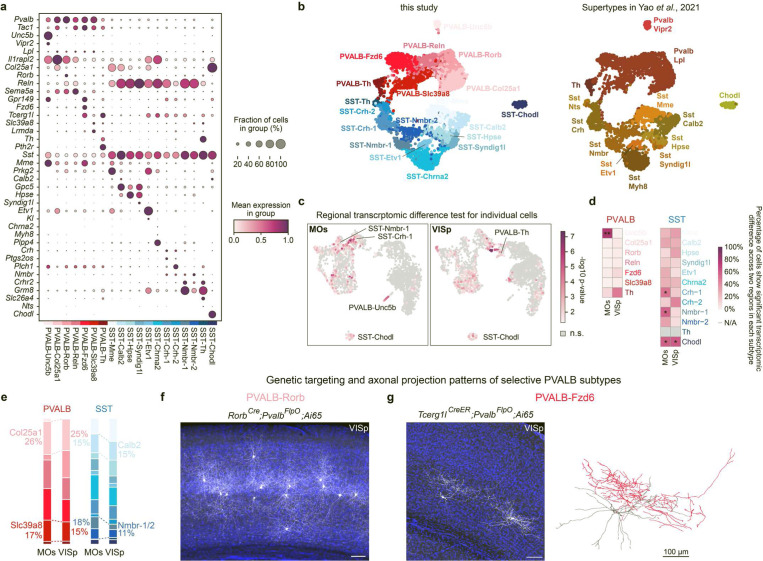
Features of PVALB and SST interneuron subtypes and regional-specific transcriptomic differences. **a,** Dot plot showing the expression of marker genes for each PVALB and SST intemeuron subtype. **b,** Analysis of published data showing the correspondence between subtypes identified in this study and supertypes defined in a previous publication^2^. **c,** Transcriptomic differences of PVALB and SST interneuron between MOs and VISp regions were assessed by Emergene (See Methods), which calculates a p-value reflecting the enrichment of region-specific gene signatures for individual nuclei, via a permutation test. n.s., not significant. **d,** Heatmap showing the percentage of cells within each cluster that exhibited significant regional differences. Cell types with ≥ 50% of cells showing regional significance were annotated with the p-value at the 50th percentile for each cell type as calculated by Emergene (*p<0.05; **p< 0.01). N/A: not analyzed, cluster size <10 cells. Detailed p-values are provided in Supplemental Table 2. **e,** Same bar plot as in Fig. 1b, now fully colored to indicate all subtypes. **f,** Intersectional genetic strategy preferentially targeting PVALB-Rorb interneurons, showing their axons concentrated in L4. Note that this strategy also labels some L5 PNs in SSp. **g,** Intersectional genetic strategy preferentially targeting PVALB-Fzd6 interneurons, showing that these interneurons reside in L5b and extend their axons laterally within L5b. Sparse labeling can be achieved via low dose of tamoxifen administration, allowing for the reconstruction of PVALB-Fzd6 interneuron morphology, with one example shown to the right. Note that this strategy also labels L5 PT neurons in SSp. Scale bars: 100 μm.

**Figure 1.**
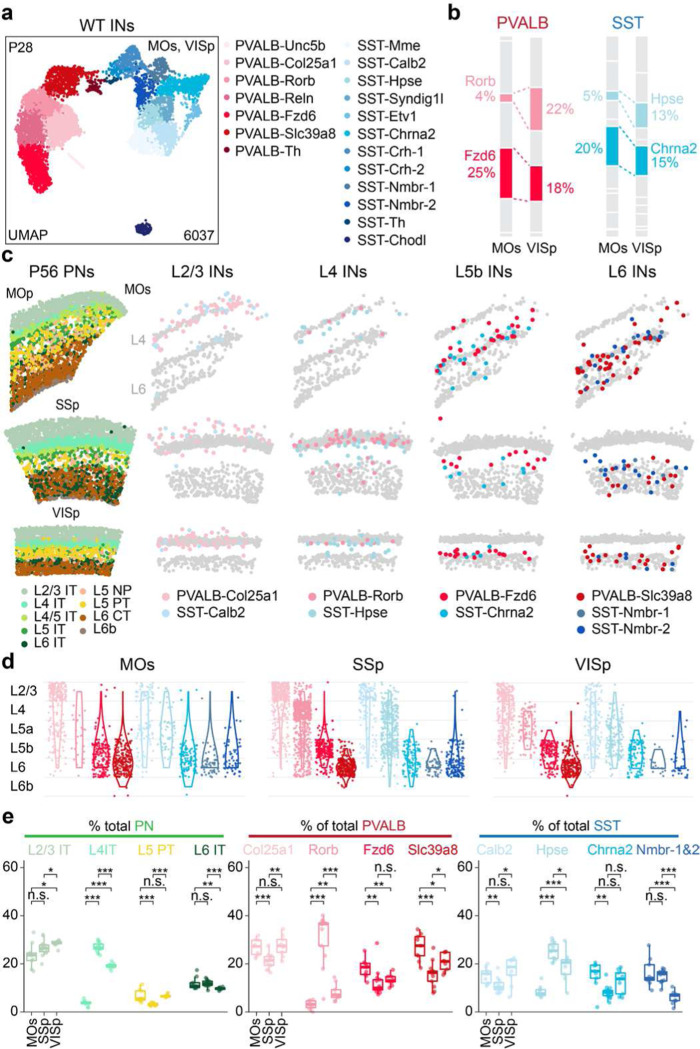
PVALB and SST interneuron subtypes show stereotyped laminar distribution and regional-specific changes in proportion. **a,** Uniform manifold approximation and projection (UMAP) of snRNA-seq data on genetically labeled cortical interneurons of P28 *Dlx5/6-Cre;Rosa26*^*LSL-h2b-GFP*^ mice, depicting 7 PVALB subtypes and 12 SST subtypes. Data was collected from both MOs (ALM) and VISp regions^17^. **b,** Proportions of individual subtypes within the total PVALB or SST population based on snRNA-seq data, compared between two sampled regions. **c,** Representative MERFISH spatial map of coronal brain sections of a P56 mouse from published datasets^18^, illustrating the distribution of different pyramidal neurons (PNs) and selected interneuron subtypes across three cortical regions. **d,** Violin plots showing the laminar distribution of selected PVALB and SST interneuron subtypes in MOs (n=9 ROIs), SSp (n=13 ROIs), and VISp (n=9 ROIs) regions based on MERFISH data. **e,** Boxplots illustrating the proportion of selected PN, PVALB, and SST subtypes found in each cortical region. Wilcoxon rank-sum test without correction for multiple comparisons, n.s. not significant, p⩾0.05; *p<0.05; **p<0.01; ***p<0.001. Detailed p-values are provided in Supplementary Table 2.

Cortical interneurons are categorized into five cardinal classes based on their expression of *Pvalb, Sst, Vip, Sncg*, or *Lamp5* genes^1–3^. The PVALB and SST classes together constitute ~70% of total cortical interneurons, while the other three classes make up the remainder. Despite recent advances in profiling the myriad cellular features of cortical interneurons, a consensus on the finer subtypes within these cardinal classes has not been reached^16^. Based on single-nuclei RNA sequencing (snRNA-seq) of postnatal day 28 (P28) mouse cortical interneurons^17^, which we believe more accurately captures the relative proportions of different interneurons than single-cell sequencing, we defined a total of 19 subtypes within the PVALB and SST interneuron classes, naming them after the marker genes they express (Fig. 1a and Extended Data Fig. 1a-b). An additional 15 subtypes were identified within the remaining three cardinal classes. However, due to their smaller size and restricted distribution mostly in L2/3, this study primarily focuses on PVALB and SST interneurons to better illustrate the principles discussed here.

To explore regional differences in the transcriptome of each interneuron subtype, we divided the snRNA-seq dataset based on the cortical regions from which interneurons were collected. All subtypes were present in both the secondary motor cortex (MOs) and the primary visual cortex (VISp), two regions located at the opposite ends of the anterior-posterior axis of the brain. We then developed a computational method to quantitatively assess the transcriptomic differences across regions that uses each cell’s nearest neighborhood as a unit and compares the closest neighborhoods across two cortical regions within an integrated transcriptomic dimensional space, assigning each cell a significance score (See Methods). Consistent with previous studies^3^, interneurons generally showed consistent transcriptomic profiles across both cortical regions, with most PVALB interneurons showing no significant regional differences. Nonetheless, minor regional transcriptomic variations were observed more prominently in SST interneurons than in PVALB interneurons (Extended Data Fig. 1c). Analyzing each subtype revealed that one PVALB subtype (PVALB-Unc5b chandelier cells) and three SST subtypes (SST-Crh-1, SST-Nmbr-1, SST-Chodl) differed significantly between the MOs and VISp regions, with more than 50% of cells showing significant regional differences (Extended Data Fig. 1d). The generally greater regional transcriptomic variation in SST interneurons likely reflects their closer association with local PNs. Consequently, the larger transcriptomic disparities in PNs across regions necessitate SST interneurons to fine-tune their gene transcription accordingly.

**Extended Data Fig. 2:**
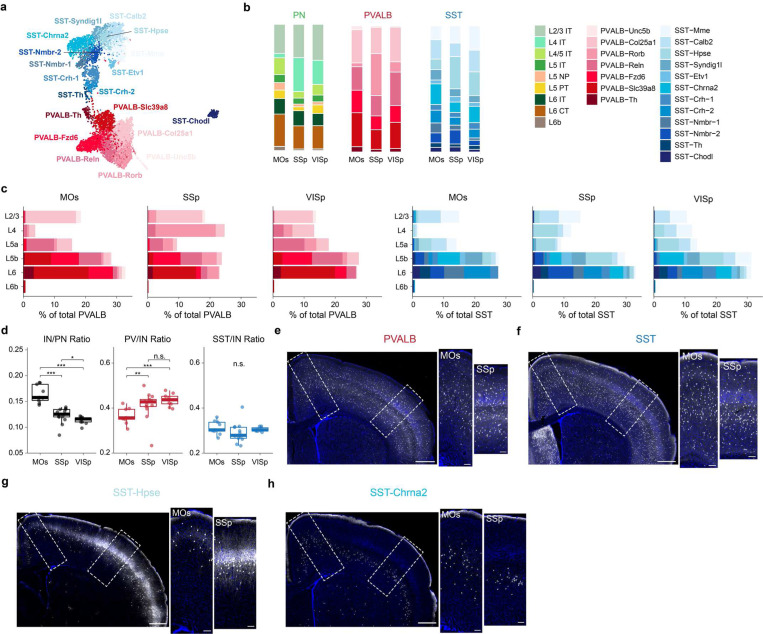
Regional differences in the proportion of different PVALB and SST interneuron subtypes. **a,** UMAP of P28 snRNA-seq data shown in Fig. 1a, now based on genes included in the MERFISH probe set, demonstrating that these interneuron subtypes can be identified based on this 500 gene set. **b,** Proportion of PN, PVALB, SST interneuron subtypes in different cortical regions based on MERFISH data. **c,** Bar plots showing the composition of PVALB and SST interneuron subtypes in different cortical layers across three cortical regions. **d,** Boxplots comparing interneuron (IN) to PN ratio, the ratio of PVALB interneurons in total interneurons, and the ratio of SST interneurons in total interneurons in different cortical regions. Wilcoxon rank-sum test, n.s. not significant, p≥0.05; *p<0.05; **p< 0.01; ***p<0.001. Detailed p-values are provided in Supplemental Table 2. **e,** Coronal brain section of a P26 mouse immunolabeled for PV (white) and counterstained by DAPI (blue). Scale bar: 500 μm. Higher magnification images of the two different cortical regions outlined are shown to the right. Scale bar: 100 μm. **f-h,** Same arrangement as in (**e**), showing genetically label SST interneurons and SST interneuron subtypes. **f,** All SST labeled in a P26 *Sst*^*Cre*^;*Sst*^*FlpO*^;*Ai65* mouse; **g,** SST-Hpse labeled in a P43 *Pdyn*^*Cre*^;*Npy*^*FlpO*^;*Ai65* mouse. Note that this strategy labels some SST-Calb2 interneurons; **h,** SST-Chrna2 labeled in a P35 *Chrna2-Cre;Ai14* mouse.

**Extended Data Fig. 3:**
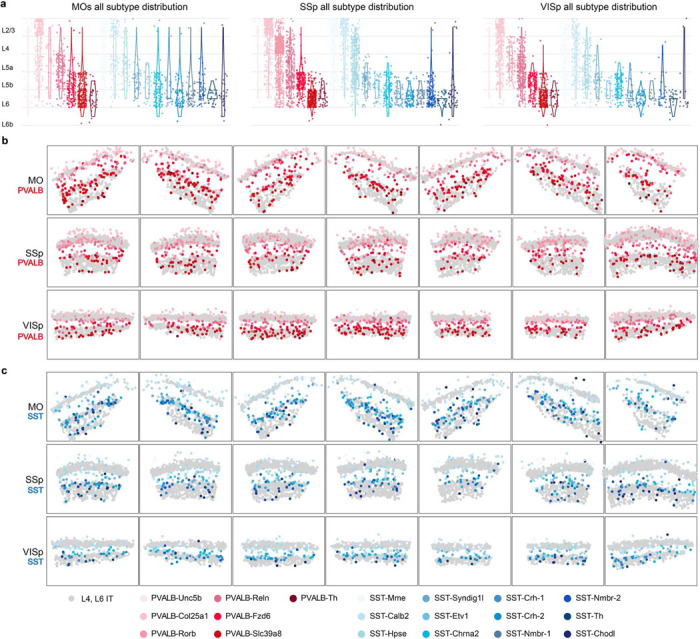
Gallery of MERFISH datasets showing the distribution of PVALB and SST interneuron subtypes. **a,** Violin plots as in Fig. 1d, now showing the distribution of all PVALB and SST subtypes across three cortical regions. **b-c,** Gallery of MERFISH spatial maps of different brain sections showing all identified PVALB and SST interneurons.

Utilizing the published adult mouse whole-brain multiplexed error-robust fluorescence *in situ* hybridization (MERFISH) dataset^18^, we examined the distribution patterns of these interneuron subtypes (Extended Data Fig. 2a). Each subtype showed a laminar specific distribution that is consistent across different cortical regions (Fig. 1c-d, Extended Data Fig. 3). In most cases, their spatial positioning correlates with the types of PN they are believed to target. For example, PVALB-Rorb and SST-Hpse both show axonal projections that preferentially target layer 4 (L4)^4^ (Extended Data Fig. 1f) and are predominantly localized within or near L4. Additionally, both PVALB-Fzd6 and SST-Chrna2 (N.B. also referred to as SST-Myh8^2,4^, Extended Data Fig. 1b) are specifically situated in L5b. The former extend their axons laterally within L5b (Extended Data Fig. 1g), while the latter project their axons to L1 but preferentially target the dendrites of L5b pyramidal tract (PT) neurons^4,19^. These findings suggest that interneuron subtypes defined at this resolution anatomically form stereotypical local microcircuitry with specific PN types.

## Interneuron subtype abundance parallel local pyramidal neuron types in different cortical regions.

Although major types of PN are shared across the isocortex^2,3^, their relative proportions vary significantly across different cortical regions. The most distinct regional difference is exemplified by a notably expanded population of L4 intratelencephalic (IT) neurons in primary sensory cortices compared to non-sensory regions (L4 IT: 3% in MOs, 27% in SSp, 19% in VISp) (Fig. 1e, Extended Data Fig. 2b). Interestingly, the proportion of interneuron subtypes in different cortical regions appears to vary according to the abundance of the PN types they target. For instance, based on the MERFISH dataset, while the overall density of SST interneurons remains comparable across different cortical regions^20^, the proportion of L4-targeting SST-Hpse more than doubles in sensory cortices compared to MOs (SST-Hpse: 8% in MOs, 25% in SSp, 19% in VISp). Similarly, the proportion of PT-targeting SST-Chrna2 interneurons^4,19^ varies across different regions (SST-Chrna2: 16% in MOs, 8% in SSp, 13% in VISp), aligning with the relative abundance of L5b PT neurons in each region (L5 PT: 7% in MOs, 3% in SSp, 7% in VISp) (Fig. 1c,e). These regional variations are also evident in snRNA-seq data and through genetic labeling of specific SST subtypes (Fig. 1b, Extended Data Fig. 1e, 2d-h). PVALB interneurons exhibit similar regional differences, with L4-targeting PVALB-Rorb being more prevalent in sensory cortices (PVALB-Rorb: 3% in MOs, 33% in SSp, 8% in VISp), while L5b PVALB-Fzd6 are more abundant in the motor cortex (PVALB-Fzd6: 18% in MOs, 11% in SSp, 13% in VISp) (Fig. 1b,c,e, Extended Data Fig. 2b). Beyond these prominent differences, other interneuron subtypes show more subtle regional variations, which nonetheless generally mirror the relative proportions of PN types. For instance, the lower proportion of L6 interneurons in VISp, PVALB-Slc39a8 and SST-Nmbr-1/2, correlates with a lower proportion of L6 IT neurons in VISp (Fig. 1e, Extended Data Fig. 1e). For a comprehensive overview, a breakdown of the proportions of each interneuron subtypes present in every layer for MOs, SSp and VISp is provided (Extended Data Fig. 2c). These findings reveal a close correspondence between interneuron subtypes and their associated PN populations. This suggests that as the interneurons migrate into the cortex during development, their interactions with local PN likely play a critical role in establishing the cell-type-specific microcircuitry observed in the adult cortex.

## Pyramidal neuron identity controls interneuron subtype composition

**Extended Data Fig. 4:**
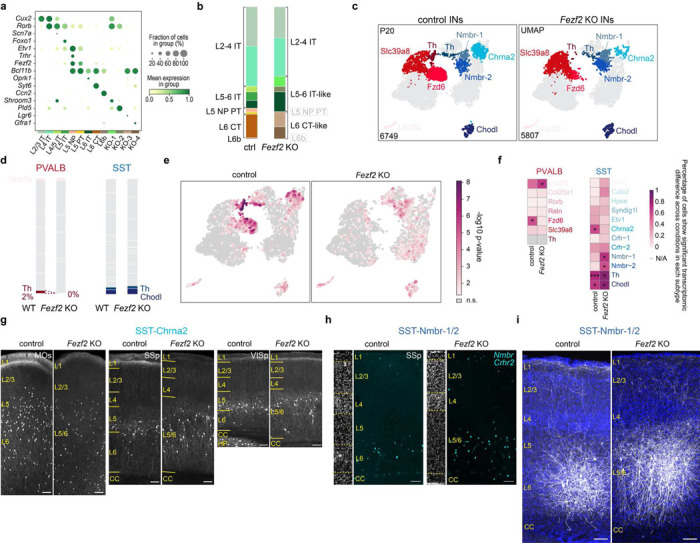
Change of cortical interneurons in *Fezf2* mutants. **a,** Dot plot showing the expression of marker genes for different PN subtypes found in control and *Fezf2* KO cortices. **b,** Bar plot as in Fig. 2c, now showing the proportion of all PN subtypes. **c,** UMAP plot as in Fig. 2d, now highlighting all subtypes that exhibited either transcriptomic or proportional changes in *Fezf2* mutants. **d,** Bar plot as in Fig. 2e, now highlighting other subtypes that show changes in *Fezf2* mutants. **e,** Transcriptomic differences of PVALB and SST interneuron between control and *Fezf2* KO conditions were assessed by Emergene (See Methods), which calculates a p-value reflecting the enrichment of genotype-specific gene signatures for individual nuclei, via a permutation test. n.s., not significant. **f,** Heatmap showing the percentage of cells within each cluster that exhibited significant regional differences. Cell types with ≥ 50% of cells showing significance between two conditions were annotated with the p-value at the 50th percentile for each cell type, as calculated by Emergene (*p<0.05; **p< 0.01). N/A: not analyzed, cluster size <10 cells. Detailed p-values are provided in Supplemental Table 2. **g,** Representative images of genetically labeled SST-Chrna2 in different cortical regions of control (*Chrna2-Cre;Fezf2*^*lacZ/+*^;*Ai14*) and *Fezf2* mutant (*Chrna2-Cre;Fezf2*^*lacZ/lacZ*^;*Ai14*) mice at P29. Scale bar: 100 μ.m. **h,** Representative RNAscope *in situ* hybridization images showing *Nmbr* and *Crhr2* mRNA transcripts, two marker genes for SST-Nmbr-1 and SST-Nmbr-2 subtypes, in control and *Fezf2* KO mice at P28. Scale bar: 100 μ.m. **i,** Representative images of labeled SST-Nmbr-1/2 interneurons in control and *Fezf2* mutants at P46. AAV-PHP.eB-hDlx-DIO-ChR2-mCherry virus was introduced to SSp of control (*Crhr2*^*Cre*^;*Fezf2*^*lacZ/+*^) and *Fezf2* mutant (*Crhr2*^*Cre*^;*Fezf2*^*lacZ/lacZ*^) mice via stereotaxic injection to label these L6 SST interneurons. Scale bar: 100 μm. CC, corpus callosum; HP, hippocampus.

**Figure 2.**
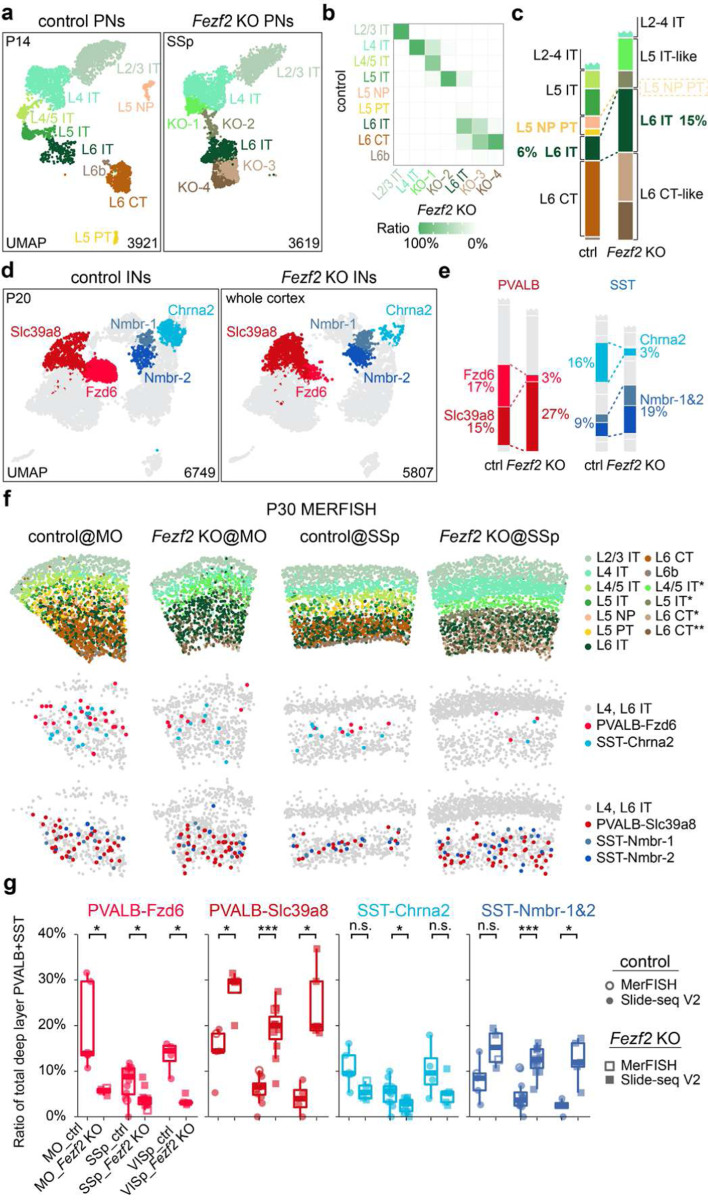
*Fezf2* KO changes the proportion of deep-layer PVALB and SST interneuron subtypes. **a,** UMAP visualization of published snRNA-seq data of excitatory neurons from P14 control (*Fezf2* HET) and *Fezf2* KO mouse SSp cortices^30^. b, Heatmap illustrating the correspondence of each PN type between control and *Fezf2* KO based on transcriptomic similarity. KO-1 through KO-4 have been named as L4/5 IT*, L5 IT*, L6 CT*, and L6 CT** thereafter to reflect their mapped identity. **c,** Proportion of deep-layer PNs in snRNA-seq data of control and *Fezf2* KO cortices. **d,** UMAP visualization of snRNA-seq data on cortical interneurons from P20 control (*Dlx5/6-Cre;Rosa26*^*LSL-h2b-GFP*^) and *Fezf2* KO (*Dlx5/6-Cre;Fezf2*^*lacZ/lacZ*^;*Rosa26*^*LSL-h2b-GFP*^) mice, highlighting five deep-layer interneuron subtypes with altered proportions in the *Fezf2* mutant. **e,** Proportion of PVALB and SST interneuron subtypes in control and *Fezf2* KO cortices based on snRNA-seq data, with selected subtypes highlighted. **f,** MERFISH spatial map of coronal brain sections from MO and SSp cortices of P30 control and *Fezf2* KO mice, illustrating the distribution of PNs and selected interneuron subtypes. **g,** Boxplot showing the proportion of selective PVALB and SST interneuron subtypes within all PVALB and SST interneurons found in L5/6, based on both MERFISH and Slide-seq data. MO_ctrl: n=5 ROIs (1 MERFISH), n=4 mice; MO_*Fezf2* KO: n=4 ROIs (1 MERFISH), n=4 mice; SSp_ctrl: n=12 ROIs (1 MERFISH), n=8 mice (including 2 mice that are *Fezf2* HET_*Bax* cHET); *SSp_Fezf2* KO: n=11 ROIs (1 MERFISH), n=8 mice (including 2 mice that are *Fezf2* KO_*Bax* cHET); VISp_ctrl: n=4 ROIs, n=4 mice; *VISp_Fezf2* KO: n=5 ROIs, n=2 mice. Age range for all samples: 4–6 weeks (including one published Slide-seq data^40^: puck 200306_02). Wilcoxon rank-sum test, n.s. not significant, p⩾0.05; *p<0.05; **p<0.0i; ***p<0.00i. Detailed p-values are provided in Supplementary Table 2.

Previous studies using various mutant models have shown that changes in PN identity can influence the overall laminar distribution of interneurons and alter inhibitory synaptic properties^21–27^. To test how changes in PN identity impact the precise relationship between specific pyramidal neuron and interneuron subtypes, we examined cortical interneurons in *Fezf2* knockout (KO) mice. *Fezf2* is a key transcription factor for the specification of deep-layer PNs and is never expressed within interneuron populations. Unlike other mutant models (e.g., *Reeler, Satb2* KO) where the transcriptomes of most PNs are significantly affected, the loss of *Fezf2* results in a clear fate-switch of subcerebral projection neurons to callosal projection neurons, offering a unique opportunity to study the changes in those interneuron subtypes that specifically associated with the two impacted populations^22,28,29^. Consistent with previous reports, analysis of published snRNA-seq data^30^ revealed that the absence of *Fezf2* resulted in a complete loss of L5 near-projecting (NP) and PT neurons, two populations that normally reside in L5b, and an increase in L6 IT neurons. Meanwhile, other pyramidal neuron types L4/5 IT, L5 IT, and L6 corticothalamic (CT) populations were mostly preserved albeit with altered transcriptomes (Fig. 2a-c, Extended Data Fig. 4a-b). Using the *Dlx5/6-Cre* driver line to enrich for interneurons, we performed snRNA-seq on interneurons from both control and *Fezf2* KO cortices of P20 mice. Interestingly, two interneuron subtypes located in L5b, PVALB-Fzd and SST-Chrna2, showed an approximately 80% reduction in *Fezf2* KO cortex. Conversely, subtypes residing in L6, including PVALB-Slc39a8, SST-Nmbr-1/2, showed an approximate two-fold increase in proportion (Fig. 2d-e). Previous studies have shown that SST-Chrna2 preferentially innervates L5 PT, whereas SST-Nmbr-1/2 preferentially innervates deep-layer IT neurons^4^. Hence, these changes match with the changes in the PN types in *Fezf2* mutant and the expected connectivity of these interneuron subtypes. Additionally, there seems to be a paradoxical loss of L6 PVALB-Th interneurons in the *Fezf2* mutant, which constitutes a small fraction in control (2% of total PVALB) and was not further investigated in this study (Extended Data Fig. 4c-d).

**Extended Data Fig. 5:**
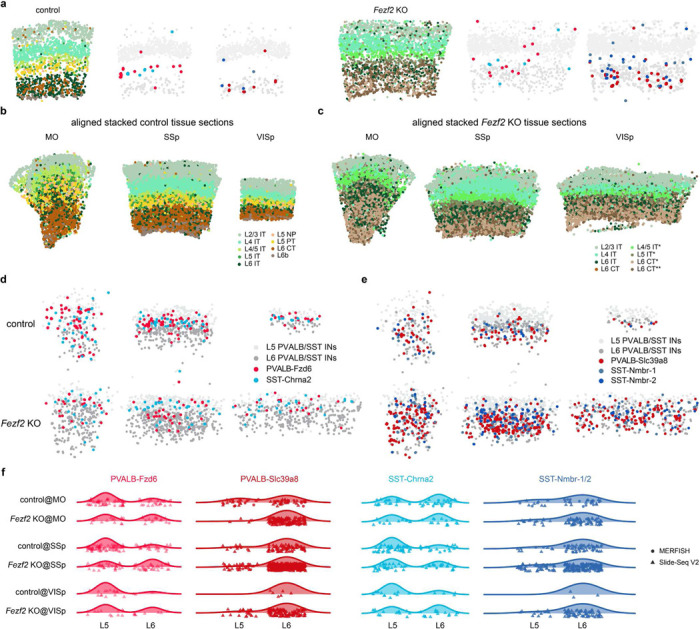
Slide-seq data demonstrating changes in cortical interneurons in *Fezf2* mutants. **a,** Representative Slide-seq data on a single coronal brain section of the SSp cortex from (left) a P37 WT mouse and (right) a P37 *Fezf2* KO mouse. **b,** Stacked Slide-seq data aligning multiple brain sections of P28–37 WT mice, illustrating the distribution of PNs. MO: n=4 ROIs, n=3 mice; SSp: n=8 ROIs, n=5 mice; VISp: n=4 ROIs, n=4 mice. **c,** Stacked Slide-seq data from 4–6 weeks old *Fezf2* KO mice. MO: n=3 ROIs, n=3 mice; SSp: n=6 ROIs, n=5 mice; VISp: n=4 ROIs, n=2 mice. **d-e,** Deep-layer PVALB and SST interneurons identified in control and *Fezf2* KO mutants (same dataset as in **b-c**), highlighting selective PVALB and SST interneuron subtypes. **f,** Ridge plots showing the distribution of selected PVALB and SST subtypes in control and *Fezf2* KO brains, demonstrating the shifted laminar location of PVALB-Fzd6 and SST-Chrna2 towards L6 in *Fezf2* mutants. The same dataset is used as in Fig. 2g.

To confirm these results, we conducted MERFISH and Slide-seq experiments, two orthogonal spatial transcriptomic methods, to examine the number and distribution of deep-layer PVALB and SST interneurons in different cortical regions of adult control and *Fezf2* KO mice (Fig. 2f; Extended Data Fig. 5a-e). These results further demonstrated the loss of L5b and gain of L6 PVALB and SST interneurons in *Fezf2* mutants^22^ that are observed across MOs, SSp and VISp regions (Fig. 2g). Moreover, the limited remaining PVALB-Fzd6 and SST-Chrna2 interneurons in *Fezf2* mutants showed a shifted distribution towards L6, whereas the expanded populations of PVALB-Slc39a8 and SST-Nmbr-1/2 interneurons had a broader distribution, although predominantly confined to the expanded L6 in the mutant cortex (Extended Data Fig. 5f). These changes in the number and distribution of SST interneurons were further confirmed by the genetic labeling of SST-Chrna2 subtype and RNAscope *in situ* hybridization against genetic markers for SST-Nmbr-1/2 subtypes in control and *Fezf2* mutants (Extended Data Fig. 4g-h).

**Extended Data Fig. 6:**
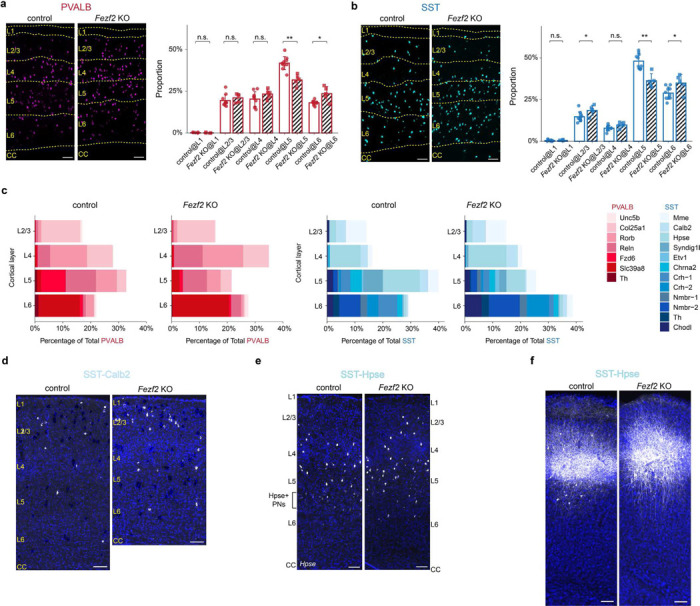
Shifted overall laminar distribution of PVALB and SST interneurons in *Fezf2* mutants. **a,** (left) Representative RNAscope *in situ* hybridization images showing labeled *PvaIb* mRNA transcripts on fixed-frozen coronal sections of P28 control and *Fezf2* KO brains in the SSp region. Scale bar: 100 μm. (right) Bar plot illustrating the proportion of PVALB interneurons found in different cortical layers, compared between control and *Fezf2* KO brains. **b,** same as in **a,** based on RNAscope *in situ* hybridization against *Sst* mRNA. control: n=10 ROIs, n=5 mice (n=1 WT, n=2 *Bax* cHET, n=1 *Fezf2* HET_*Bax* cHET), age P26–28, n=3932 total PVALB interneurons, n=2280 total SST interneurons quantified. *Fezf2* KO: n=5 ROIs, n=2 mice (n=1 *Fezf2* KO, n=1 *Fezf2* KO_*Bax* cHET), n=2377 total PVALB interneurons, n=1677 SST interneurons quantified, age P27–28. Wilcoxon rank-sum test, n.s. not significant, p≥0.05; *p<0.05; **p< 0.01. Detailed p-values are provided in Supplemental Table 2. **c,** Bar plots showing the composition of each PVALB and SST interneuron subtypes in different cortical layers of control and *Fezf2* KO brains in the SSp region, based on MERFISH dataset. control: n=3 ROIs, n=2 mice (n=1 WT, n=1 *Bax*_cHET), age: P14–30. *Fezf2* KO: n=3 ROIs, n=3 mice (n=2 *Fezf2* KO, n=1 *Fezf2* KO_*Bax* cHET), age: P14–30. **d,** Representative RNAscope *in situ* hybridization images showing SST-Calb2 interneurons, showing no obvious shift in the laminar distribution of these interneurons in *Fezf2* KO mouse cortices in the SSp region. The signal was shown as the subtraction of *Vip* signal from *Calb2* signal. Note that this strategy also labels SST-Chodl interneurons. **e,** Representative RNAscope *in situ* hybridization images showing labeled *Hpse* mRNA transcripts on fixed-frozen coronal sections of P31 control and *Fezf2* KO brains in the SSp region, demonstrating a laminar shift of SST-Hpse interneurons to more superficial layers. Note that L5 PNs express a low level of *Hpse* gene. Scale bar: 100 μm. **f,** Representative images of labeled SST-Hpse interneurons in control and *Fezf2* mutants at P47. AAV-PHP.eB-hDlx-DIO-ChR2-mCherry virus was introduced to the SSp of control (*Hpse*^*Cre*^;*Fezf2*^*lacZ/+*^) and *Fezf2* mutant (*Hpse*^*Cre*^;*Fezf2*^*lacZ/lacZ*^) mice via stereotaxic injection to label these SST interneurons. Scale bar: 100 μm.

Previous studies have shown a shift in the overall laminar distribution of PVALB and SST interneurons towards upper layers (L2/3, L4) in the *Fezf2* KO cortex ^22^. Our results corroborate this observation (Extended Data Fig. 6a-b). A close examination of the subtype composition in each cortical layer based on MERFISH data revealed that the increase in upper layer interneurons appears to result from the redistribution of PVALB-Reln and SST-Hpse subtypes, without an obvious change in their overall number (Extended Data Fig. 6c-f). Both subtypes normally reside above L6, so the expanded L6 in *Fezf2* mutants likely generates a stronger repulsive signal that pushes these interneurons further into the upper layers. This intriguing phenomenon merits further investigation, but it falls outside the primary focus of this study.

Analysis of transcriptomic changes in *Fezf2* mutants revealed that both PVALB-Fzd6 and SST-Chrna2 intemeurons lost many of their subtype-specific defining features under mutant conditions, likely due to improper maturation in the absence of their normally associated PN types. Conversely, SST-Nmbr-1/2 interneurons acquired novel features in *Fezf2* mutants (Extended Data Fig. 4e-f). Using viral genetic strategies to label these L6 SST interneurons, we observed that they seemed to have more elongated dendrites along the cortical column, and fewer L1 axons in the mutant cortex compared to the control (Extended Data Fig. 4i). These morphological changes likely reflect the transcriptomic alterations as an adaptation to the expanded L6 in the *Fezf2* mutants. In contrast, L6 PVALB-Slc39a8 interneurons did not show significant transcriptomic differences between control and mutant cortex. Akin to the lack of transcriptomic variation in PVALB interneurons across different cortical regions, these results further suggest that PVALB interneurons are less sensitive to subtle changes in the transcriptome of the PN types they are associated with, except for changes that affect PN identity.

In summary, our study of *Fezf2* mutants, which solely affect PN identity, revealed that PNs exert a non-autonomous, subtype-specific influence on cortical interneurons. These findings demonstrate that the relative numbers and distributions of different interneuron subtypes depend significantly on the specific PN subtypes to which they are ultimately connected.

## Preventing *Bax*-dependent apoptosis partially rescues the *Fezf2* mutant phenotype on cortical interneurons

**Extended Data Fig. 7:**
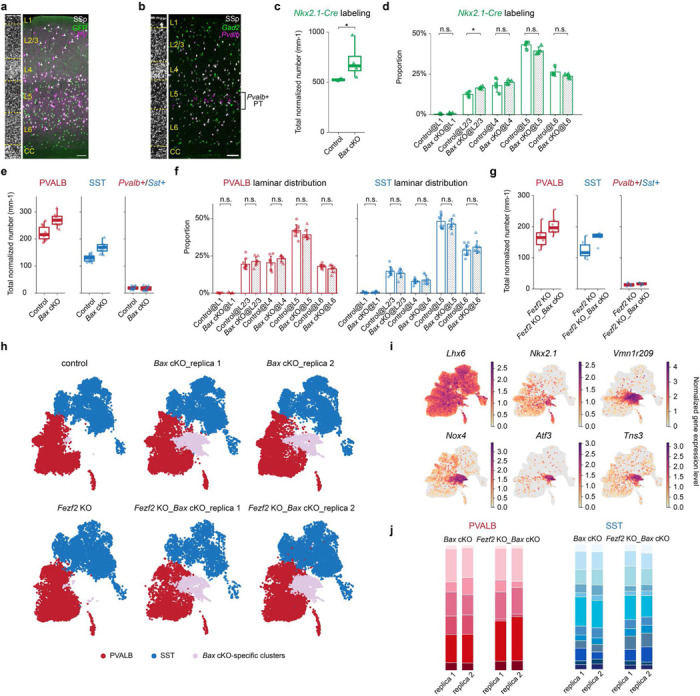
Conditional removal of *Bax* in PVALB and SST interneurons increases their number. **a,** Representative image of coronal brain sections from a P26 *Nkx2.1-Cre;Bax*^*fl/+*^;*Rosa26*^*LSL-h2b-GFP*^ mouse, immunostained against GFP and PV, illustrating the incomplete labeling of superficial PV interneurons in the SSp region. Scale bar: 100 μ.m. **b,** Representative RNAscope *in situ* hybridization images showing labeled *Pvalb* and *Gad2* mRNA transcripts, highlighting the expression of *Pvalb* gene outside of interneurons, specifically in *Gad2-* L5b PT neurons in the SSp region. Scale bar: 100 μ.m. **c,** Quantification of genetically labeled interneurons by *Nkx2.1-Cre;Rosa26*^*LSL-h2b-GFP*^ in control and *Bax* cKO conditions. control: n=3 mice (n=2 *Bax* cHET, n=2 *Fezf2* HET_*Bax* cHET), n=2316 GFP+ interneurons counted; *Bax* cKO: n=4 mice (n=2 *Bax* cKO, n=2 *Fezf2* HET_*Bax* cKO), n=3548 GFP+ interneurons counted. **d,** Laminar distribution of genetically labeled interneurons compared between control and *Bax* cKO conditions. Same dataset as in **c.** Error bars show standard deviations. **e,** Quantification of PVALB and SST interneurons in control and *Bax* cKO cortices in the SSp region, based on results from RNAscope *in situ* hybridization of fixed-frozen brain sections. L5 PT neurons were identified as *Pvalb+/Lhx6-* cells located in L5b and subsequently excluded from the quantification. Numbers are normalized to the length of the outskirts of the cortex in millimeters. control: n=10 ROIs, n=5 mice (n=1 WT, n=2 *Bax* cHET, n=1 *Fezf2* HET_*Bax* cHET), age P26–28, n=3932 total PVALB interneurons, n=2280 total SST interneurons, n=337 *Pvalb+/Sst+* interneurons. *Bax* cKO: n=8 ROIs, n=4 mice (n=2 *Bax* cKO, n=2 *Fezf2* HET_*Bax* cKO), age P26–28, n=3470 total PVALB interneurons, n=2150 total SST interneurons, n=221 total *Pvalb+/Sst+* interneurons quantified. **f,** Same dataset as in **d,** showing the distribution of PVALB and SST interneurons in different cortical layers was not altered in *Bax* cKO. Error bars show standard deviations. **g.** Quantification of PVALB and SST interneurons in *Fezf2* KO and *Fezf2* KO_*Bax* cKO cortices, based on RNAscope *in situ* hybridization of fresh-frozen sections. *Fezf2* KO: n=6 ROIs, n=4 mice (n=1 *Fezf2* KO, n=3 *Fezf2* KO_*Bax* cHET), age P28–33, n=1668 total PVALB interneurons, n=1257 total SST interneurons, n=138 total *Pvalb+/Sst+* interneurons quantified. *Fezf2* KO_*Bax* cKO: n=6 ROIs, n=4 mice, age P28–33, n=2161 total PVALB interneurons, n=1768 total SST interneurons, n=175 total *Pvalb+/Sst+* interneurons quantified. **h,** UMAP plots of P14 snRNA-seq data on interneurons from four different genotypes, showing a group of cells that are specific to the *Bax* cKO and *Fezf2* KO_*Bax* cKO conditions. **i,** UMAP plots showing the normalized gene expression level of selected genes that are expressed in *Bax* cKO-specific clusters. **j,** Stacked bar plots showing that the proportion of different PVALB and SST interneurons remains relatively consistent across biological replicates of snRNA-seq of cortical interneurons from *Bax* cKO and *Fezf2* KO_*Bax* cKO brains. **d** and **f,** Wilcoxon rank-sum test, n.s. not significant, p≥0.05; *p<0.05. Detailed p-values are provided in Supplemental Table 2.

**Figure 3.**
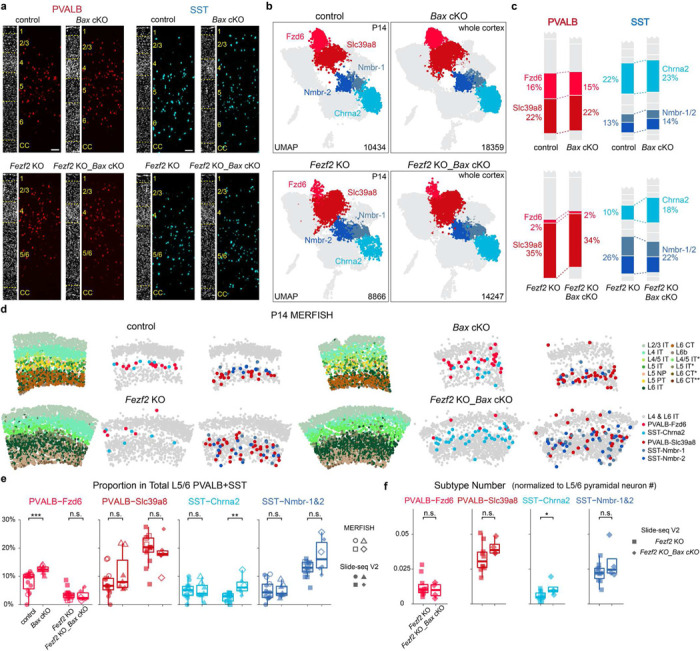
Preventing cell death rescues the loss of SST-Chrna2 but not PVALB-Fzd6 interneurons in *Fezf2* mutants. **a,** Representative RNAscope *in situ* hybridization images showing labeled *Pvalb* and *Sst* mRNA transcripts in the SSp region of P28–33 mice with four different genotypes. DAPI counterstaining is provided on the left of each image for laminar distribution reference. Scale bar: 100 μm. **b,** UMAP visualization of snRNA-seq of sorted interneurons from control (*Nkx2.1-Cre;Bax*^*fl/+*^;*Rosa26*^*LSL-h2b-GFP*^), *Bax* cKO (*Nkx2.1-Cre;Bax*^*fl/fl*^;*Rosa26*^*LSL-h2b-GFP*^*), Fezf2* KO (*Nkx2.1-Cre;Fezf2*^*lacZ/lacZ*^;*Rosa26*^*LSL-h2b-GFP*^), and *Fezf2* KO_*Bax* cKO (*Nkx2.1-Cre;Fezf2*^*lacZ/lacZ*^;*Bax*^*fl/fl*^;*Rosa26*^*LSL-h2b-GFP*^) mice at P14. control: n=1, *Bax* cKO: n=2, *Fezf2* KO: n=1, *Fezf2* KO_*Bax* cKO: n=4 mice. **c,** Proportion of deep-layer PVALB and SST interneuron subtypes in snRNA-seq dataset. **d,** MERFISH spatial map of coronal brain sections from SSp region of P14 mice of the four genotypes, showing PN and selected interneuron subtypes. **e,** Proportion of selected PVALB and SST interneuron subtypes within all PVALB+SST interneurons found in L5/6, based on spatial transcriptomic data in the SSp region. Control: n=14 ROIs (3 MERFISH), n=10 mice (WT: n=7, *Bax* cHET: n=1, *Fezf2* HET_*Bax* cHET: n=2); *Bax* cKO: n=7 ROIs (2 MERIFSH), n=4 mice (all *Fezf2* HET_*Bax* cKO); *Fezf2* KO: n=13 ROIs (3 MERFISH), n=10 mice (*Fezf2* KO: n=7, *Fezf2* KO_*Bax* cHET: n=3); *Fezf2* KO_*Bax* cKO: n=6 ROIs (2 MERFISH), n=4 mice. Age range: P14–37, with one *Fezf2* KO mouse at 6-weeks old. **f,** Number of selected PVALB and SST subtypes normalized to the number of deep-layer PNs based on Slide-seq data. *Fezf2* KO: n=10 ROIs, n=7 mice (*Fezf2* KO: n=5, *Fezf2* KO_*Bax* cHET: n=2); *Fezf2* KO *Bax* _ cKO: n=4 ROIs, n=3 mice. Wilcoxon rank-sum test, n.s. not significant, p≥0.05; *p<0.05; **p<0.01; ***p<0.001. Detailed p-values are provided in Supplementary Table 2.

Previous studies have shown that ~40% of cortical interneurons undergo programmed cell death around P7–9, a process modulated by PNs^31–33^. To directly test whether PNs influence the relative number of different interneuron subtypes by modulating their survival, we prevented cell death in PVALB and SST interneurons by removing *Bax*, a key gene involved in apoptosis of cortical interneurons, using *Nkx2.1-Cre;Bax*^*fl/fl*^. To confirm the effects of *Bax* removal, we quantified genetically labeled cortical interneurons in control (*Nkx2.1-Cre;Bax*^*fl/+fl*^;*Rosa26*^*LSL-h2b-GFP*^) and *Bax* cKO (*Nkx2.1-Cre;Bax*^*fl/fl*^;*Rosa26*^*LSL-h2b-GFP*^) mice and observed a 36% increase in the total amount of labeled interneurons in *Bax* mutants (Extended Data Fig. 7c-d). Specifically, RNAscope *in situ* hybridization experiments showed a 22% increase of PVALB interneurons and a 31% increase of SST interneurons in *Bax* cKO, without affecting their laminar distribution (Fig. 3a, Extended Data Fig. 7b,e-f). Consistent with previous reports suggesting no significant increase in interneuron cell death in *Fezf2* mutant^22^, *Fezf2* KO *Bax* cKO mice (*Nkx2.1-Cre;Fez2*^*lacZ/lacZ*^;*Bax*^*fl/fl*^) showed a 22% increase in PVALB interneurons and a 32% increase in SST interneurons compared to *Fezf2* KO mice, mirroring the increase observed in control mice (Fig. 3a, Extended Data Fig. 7g).

To compare the effects of *Bax* removal on the composition of cortical intemeurons in both control and *Fezf2* mutant conditions, we performed snRNA-seq of PVALB and SST intemeurons from control, *Bax* cKO, *Fezf2* KO, and *Fezf2* KO_*Bax* cKO mice at P14, an age when apoptosis in interneurons has concluded in the control condition. In both *Bax* cKO conditions, we observed two clusters, constituting ~10% of the total interneurons in the dataset, that were absent in both the control and *Fezf2* KO conditions. These *Bax* cKO-specific interneurons expressed genes typically found in interneurons outside the cortex (Extended Data Fig. 7h-i), suggesting they are likely misguided interneurons, and were not further analyzed. The remaining interneurons across all four conditions aligned and integrated well (Fig. 3b). To account for a potential increased variability in *Bax* cKO samples, we included two biological replicates for both *Bax* cKO and *Fezf2* KO_*Bax* cKO conditions, and the results seemed consistent across replicates (Extended Data Fig. 7j). We then compared the proportions of different interneuron subtypes across different conditions. The proportions of interneurons in *Bax* cKO appeared comparable to control conditions, suggesting that a similar proportion of cell death occurs for each interneuron subtype during development. Interestingly, *Bax* cKO in the *Fezf2* mutant background partially rescued the loss of SST-Chrna2 but not PVALB-Fzd6 (Fig. 3c).

**Extended Data Fig. 8:**
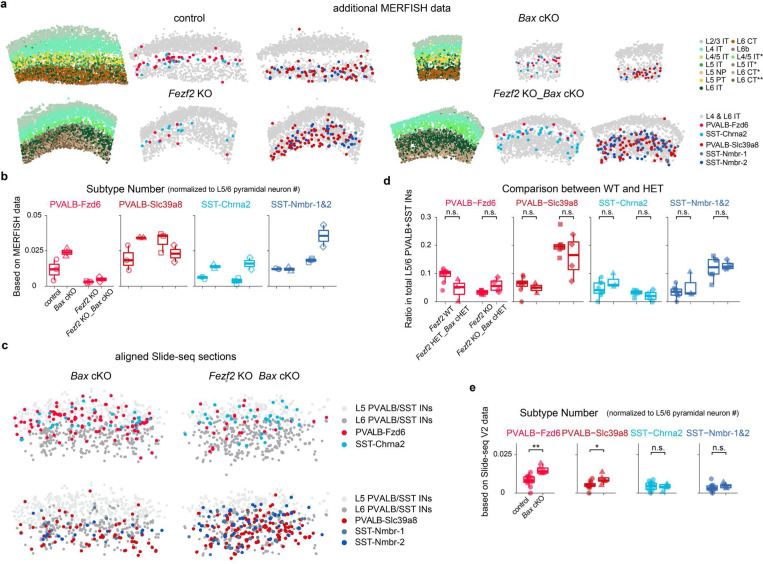
Spatial transcriptomics data showing the effects of selective removal of *Bax* in PVALB and SST interneurons on the interneuron phenotypes of *Fezf2* mutants. **a,** Additional P14 MERFISH data of different genotypes not included in Fig 3d. **b,** Number of selected PVALB and SST subtypes normalized to the number of L5/6 PNs in four different genotypes based on MERFISH data in the SSp region. No statistical test was applied due to the small dataset size. **c,** Aligned and stacked Slide-seq data on coronal brain sections from *Bax* cKO and *Fezf2* KO*_Bax* cKO mice, highlighting selected PVALB and SST interneuron subtypes identified in L5/6. *Bax* cKO: n=5 ROIs, n=3 mice; *Fezf2* KO_*Bax* cKO: n=4, n=3 mice. Age range: P28–33. **d,** Ratio of selected PVALB and SST interneuron subtypes within total L5/6 PVALB and SST interneurons are consistent between wildtype and heterozygous genotype in the SSp region, based on Slide-seq data. *Fezf2* WT: n=8 ROIs, n=5 mice; *Fezf2* HET_*Bax* cHET: n=3 ROIs, n=2 mice; *Fezf2* KO: n=6 ROIs, n=5 mice; *Fezf2* KO_*Bax* cHET: n=4 ROIs, n=2 mice. Age range: 4–6 weeks old. These data are included in Fig. 2g. **e,** Number of selected PVALB and SST subtypes normalized to the number of L5/6 PNs compared between control and *Bax* cKO. Control: n=1 1 ROIs, n=7 mice; *Bax* cKO: n=5 mice. Age range: P28–37. For **b** and **e,** Wilcoxon rank-sum test. n.s. not significant, p≥0.05; *p<0.05; **p< 0.01; ***p<0.00i. Detailed p-values are provided in Supplemental Table 2.

To confirm these results, we performed MERFISH and Slide-seq experiments across four conditions at P14 in adult mouse SSp cortices (Fig. 3d, Extended Data Fig. 8a,f). Previous studies suggested that interneuron numbers and distribution stabilize after P14^20^. As such, data from both ages were combined to increase statistical power. Additionally, no obvious differences were noted between *Fezf2* wild-type (WT) and *Fezf2* heterozygous (HET), or between *Fezf2* KO and *Fezf2* KO_*Bax* cHET (Extended Data Fig. 8d). Therefore, our control dataset included both wild-type and heterozygous alleles. Note that the *Nkx2.1-Cre* allele labels the majority of deep-layer PVALB and SST interneurons, but misses ~40–50% of those in the superficial layers^34^ (Extended Data Fig. 7a). Nevertheless, as the analysis involving *Bax* cKO conditions were focused on interneurons found in deep layers (L5–6), the lack of labeling in superficial layers is not relevant. Consistent with snRNA-seq results, *Bax* cKO in control conditions does not largely alter the proportion of interneuron subtypes, except for PVALB-Fzd6, which increased slightly from 8% in control to 12% in *Bax* cKO (Fig. 3e, Extended Data Fig. 8e). Notably, removing *Bax* in *Fezf2* mutants does not rescue the reduced proportion of PVALB-Fzd6 compared to control, while the proportion of SST-Chrna2 doubled in *Fezf2* KO_*Bax* cKO condition, reaching a level comparable to the control condition (Fig. 3e). More precisely, comparing the number of individual subtypes by normalizing to deep-layer PN numbers also showed a two-fold increase in the number of SST-Chrna2 interneurons in *Fezf2* KO*_Bax* cKO, whereas other subtypes remain unchanged with or without *Bax* in *Fezf2* mutants (Fig. 3f, Extended Data Fig. 8b).

Taken together, these results suggest that the loss of SST-Chrna2 in *Fezf2* mutants is due to increased apoptosis in this subtype, although the exact cause remains unclear. It is possible that without their associated PNs, SST-Chrna2 interneurons fail to mature properly or exhibit decreased activity, both of which could lead to programmed cell death. In contrast, the loss of PVALB-Fzd6 appears to be independent of cell death. This suggests that PNs may directly influence the differentiation of PVALB subtypes, causing PVALB-Fzd6 to transform into PVALB-Slc39a8 in *Fezf2* mutants.

## Pyramidal neurons direct PVALB subtype differentiation and promote SST subtype maturation

**Extended Data Fig. 9:**
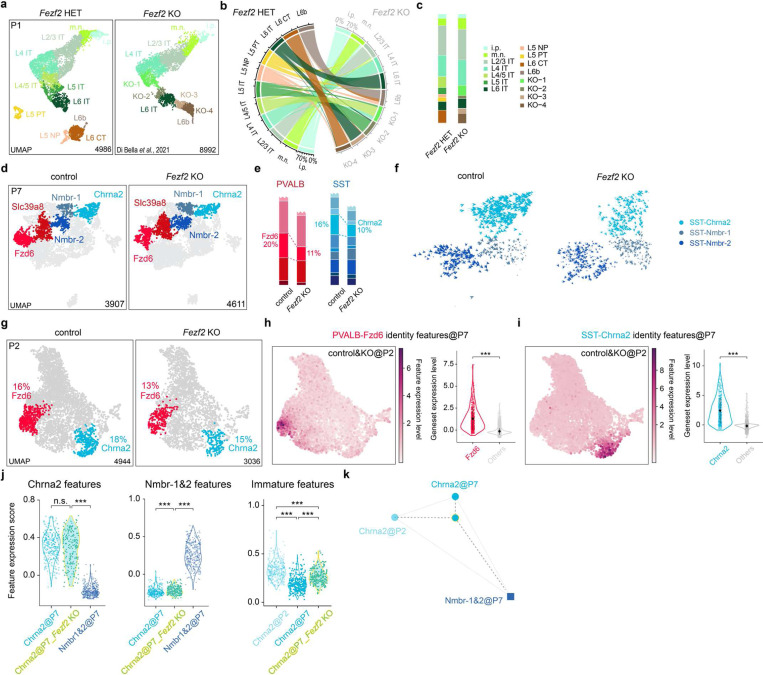
Changes in interneurons in *Fezf2* mutant at early developmental ages **a,** UMAP visualization of published snRNA-seq data^41^ of excitatory neurons from Pi *Fezf2* HET and *Fezf2* KO mouse cortices. **b,** River plot illustrating the correspondence of each PN type between Pi *Fezf2* HET and *Fezf2* KO conditions based on transcriptomic similarity, showing the proportion of mapped PN cell types within each cell type in *Fezf2* KO. **c,** Stacked bar plots showing the composition of excitatory neuron types in *Fezf2* HET and *Fezf2* KO brains based on snRNA-seq data. **d,** UMAP visualization of snRNA-seq data of cortical interneurons collected from control (*Dlx5/6-Cre;Fezf2*^*lacZ/+*^ ;*Rosa26*^*LSL-h2b-GFP*^) and *Fezfi* KO (*Dlx5/6-Cre;Fezf2*^*lacZ/lacZ*^;*Rosa26*^*LSL-h2b-GFP*^) mouse brains at P7, with selected PVALB and SST interneuron subtype highlighted. **e,** Stacked bar plots showing the proportion of deep-layer PVALB and SST subtypes in control and *Fezf2* KO brains based on snRNA-seq data. **f,** RNA velocity analysis of snRNA-seq data on cortical interneurons collected from control (*Fezf2* HET) and *Fezf2* KO mouse brains at P7, showing gene expression dynamics of three SST interneuron subtypes. **g,** UMAP visualization of snRNA-seq data of cortical intemeurons collected from control (*Nkx2.1-Cre;Fezf2*^*lacZ/+*^;*Bax*^*fl/+*^;*Rosa26*^LSL-h2b-GFP^) and *Fezf2* KO (*Nkx2.1-Cre;Fezfì*^*lacZ/lacZ*^;*Bax*^*fl/+*^;*Rosa26*^*LSL-h2b-OFP*^) mouse brains at P2, with PVALB-Fzd6 and SST-Chrna2 interneuron subtypes highlighted. **h-i,** Feature genes identified in the P7 snRNA-seq dataset that are selectively expressed in PVALB-Fzd6 and SST-Chrna2 interneuron subtypes are also selectively expressed at P2. (left) UMAP plot showing the expression level of feature genes. (right) Violin plot comparing the expression level of feature genes in a particular cluster versus the rest of the nuclei. **j,** The expression score of gene sets characteristic of SST-Chrna2, SST-Nmbr-1&2 at P7, and SST-Chrna2 at P2 (referred to as Chrna2 feature, Nmbr-1&2 feature, and immature features, respectively; see Methods) were compared between different groups of SST intemeurons. **k,** Triangular Affinity Map of SST-Chrna2 intemeurons in *Fezf2* mutants at P7 (see Methods), showing the relative transcriptomic similarities among SST-Chrna2 at P7, SST-Nmbr-1&2 at P7, and SST-Chrna2 at P2.

To find evidence for the transformation of L5b into L6 PVALB interneurons in *Fezf2* mutants, and to further elucidate the cause of increased cell death of SST-Chrna2 interneurons under such conditions, we performed snRNA-seq on cortical interneurons from control and *Fezf2* KO mice at P7. Characterizing them at the onset of programmed cell death allowed us to capture PVALB-Fzd6 in the process of transforming into PVALB-Slc39a8 and to examine the status of SST-Chrna2 interneurons as they begin to undergo apoptosis in *Fezf2* KO cortices. Even though a decrease in the number of both PVALB-Fzd6 and SST-Chrna2 interneurons was already apparent at P7 in *Fezf2* KOs (Extended Data Fig. 9d-e), these populations were sufficiently large at this age to allow for meaningful analysis before their numbers declined further.

**Extended Data Fig. 10:**
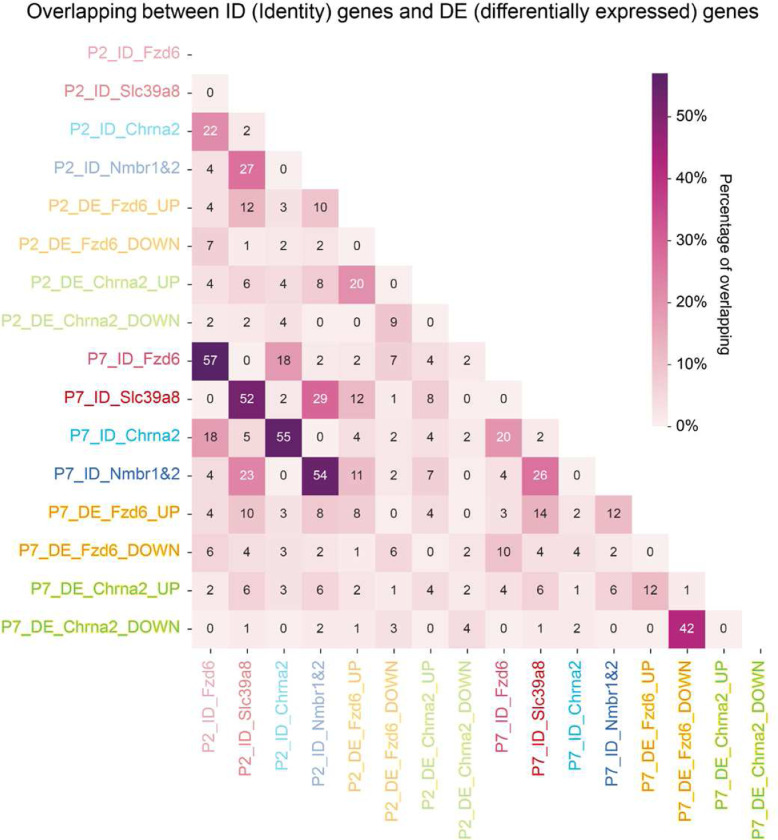
Heatmap showing the percentage of overlap between different gene sets.

**Figure 4.**
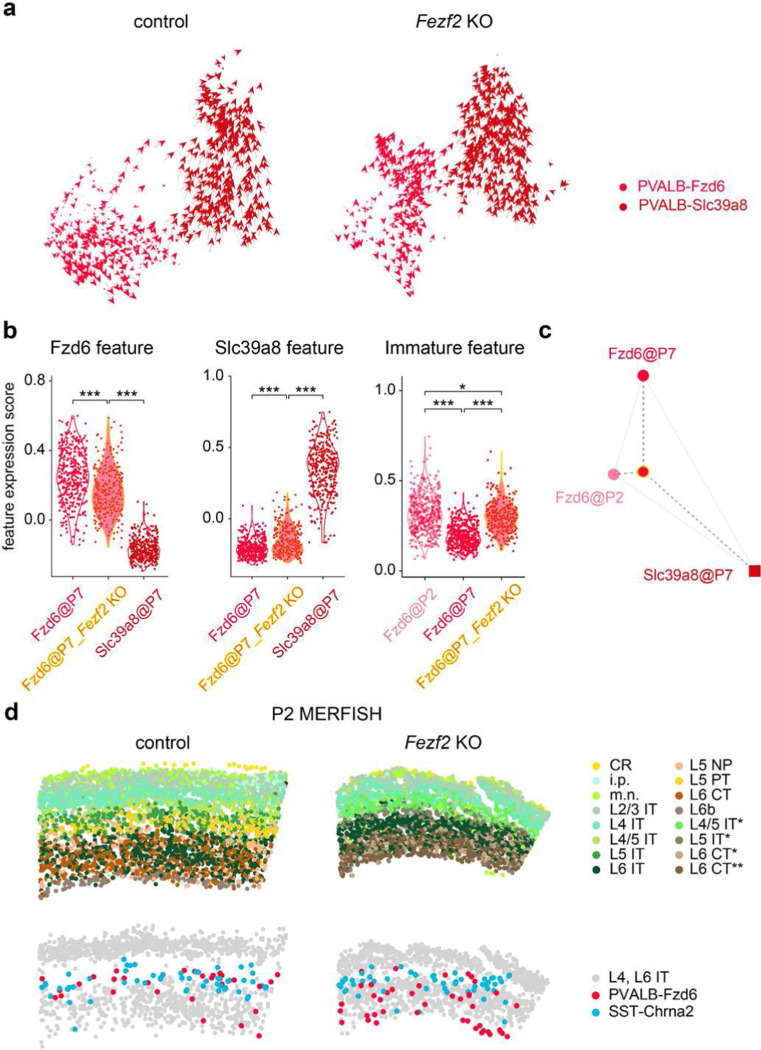
PVALB and SST interneurons in *Fezf2* mutants at early postnatal ages **a,** RNA velocity analysis of snRNA-seq data on cortical intemeurons collected from P7 control (*Fezf2* HET) and *Fezf2* KO mouse brains, showing gene expression dynamics of PVALB-Fzd6 and PVALB-Slc39a8 intemeurons. **b,** The expression score of gene sets characteristic of PVALB-Fzd6, PVALB-Slc39a8 at P7, and PVALB-Fzd6 at P2 (referred to as Fzd6 feature, Slc39a8 feature, and immature features, respectively; see Methods) were compared between different groups of PVALB intemeurons. This comparison demonstrates changes in the expression of these gene features in PVALB-Fzd6 intemeurons in *Fezf2* mutants at P7. Wilcoxon rank-sum test, *p<0.05; **p<0.01; ***p<0.001. Detailed p-values are provided in Supplementary Table 2. **c,** Triangular Affinity Map of PVALB-Fzd6 intemeurons in *Fezf2* mutants at P7 (see Methods), depicting the relative transcriptomic similarities among PVALB-Fzd6, PVALB-Slc39a8 at P7, and PVALB-Fzd6 at P2. **d,** MERFISH spatial map of coronal brain sections from the SSp region of control (*Nkx2.1-Cre;Fezf2*^*lacZ/+*^;*Bax*^*fl/+*^) and *Fezf2* KO (*Fezf2*^*lacZ/lacZ*^;*Bax*^*fl/fl*^) mice at P2, showing PN and selected interneuron subtypes. CR, Cajal-Retzius cells; i.p., intermediate progenitor; m.n., migrating neurons.

To predict how the transcriptome of each subtype will evolve developmentally, we conducted RNA velocity^35^ analysis on P7 snRNA-seq data. This analysis leverages the variations in gene expression profiles of individual cells within each subtype, which likely reflect their varying positions along the developmental trajectory, to forecast the direction of overall transcriptomic changes. Interestingly, while PVALB-Fzd6 and PVALB-Slc39a8 interneurons in the control dataset are predicted to become more distinct from each other, a major fraction of PVALB-Fzd6 interneurons in *Fezf2* mutants appear to be transitioning towards PVALB-Slc39a8 identity (Fig. 4a). In comparison, SST-Chrna2 did not show a clear trend towards SST-Nmbr-1/2 in either condition (Extended Data Fig. 9f). Using the control data as a reference, we identified marker gene sets (ID genes) that are differentially expressed between PVALB-Fzd6 and PVALB-Slc39a8, as well as between SST-Chrna2 and SST-Nmbr1/2 subtypes. Aligned with a fate switch of PVALB-Fzd6 interneurons, the expression of PVALB-Fzd6 ID genes is significantly reduced, with an increase in the expression of PVALB-Slc39a8 ID genes in these cells in *Fezf2* mutants (Fig. 4b). In contrast, SST-Chrna2 ID genes were retained in SST-Chrna2 interneurons in *Fezf2* mutants, although there was also an increased expression of genes preferentially expressed in SST-Nmbr-1/2 interneurons (Extended Data Fig. 9j). A closer examination showed that ~50% of the acquired SST-Nmbr-1/2 features in SST-Chrna2 interneurons in *Fezf2* mutants were common signatures shared between L6 PVALB and SST interneurons (Extended Data Fig. 10), likely caused by the shifted distribution of these interneurons into L6 and their adaptation to the altered local environment in *Fezf2* KOs.

As PVALB-Fzd6 interneurons in *Fezf2* mutants lose some of their defining features, we reasoned that they might remain immature without their normal synaptic partners. To test this, we compared PVALB-Fzd6 interneurons in *Fezf2* mutants at P7 to PVALB-Fzd6 in control at P2. Although not all interneuron subtypes can be confidently identified at P2 in control mice, both PVALB-Fzd6 and SST-Chrna2 are among the earliest subtypes to mature and therefore can be clearly identified at this age. Indeed, marker genes identified at P7 are already specifically expressed in these two subtypes by P2 (Extended Data Fig. 9g-i). Moreover, cross-age comparison revealed that PVALB-Fzd6 interneurons in the mutant condition retain significantly higher expression of features characteristic of an earlier developmental age (Fig. 4b). Their overall transcriptomic profile resembles P2 PVALB-Fzd6 more than P7 in control mice (Fig. 4c). In comparison, while SST-Chrna2 interneurons also showed increased immature features at P7 in *Fezf2* mutants, their overall transcriptomic profile was closer to P7 controls than P2 (Extended Data Fig. 9j-k). Finally, despite changes in PN identity in *Fezf2* mutants are already obvious at P1 (Extended Data Fig. 9a-c), there is no significant loss of either PVALB-Fzd6 and SST-Chrna2 interneurons in *Fezf2* mutants at P2, based on both snRNA-seq and MERFISH data (Fig. 4d). This suggests that changes in interneuron subtype composition in *Fezf2* mutants occur after migrating interneurons invade the cortex and interact with PNs.

**Figure 5.**
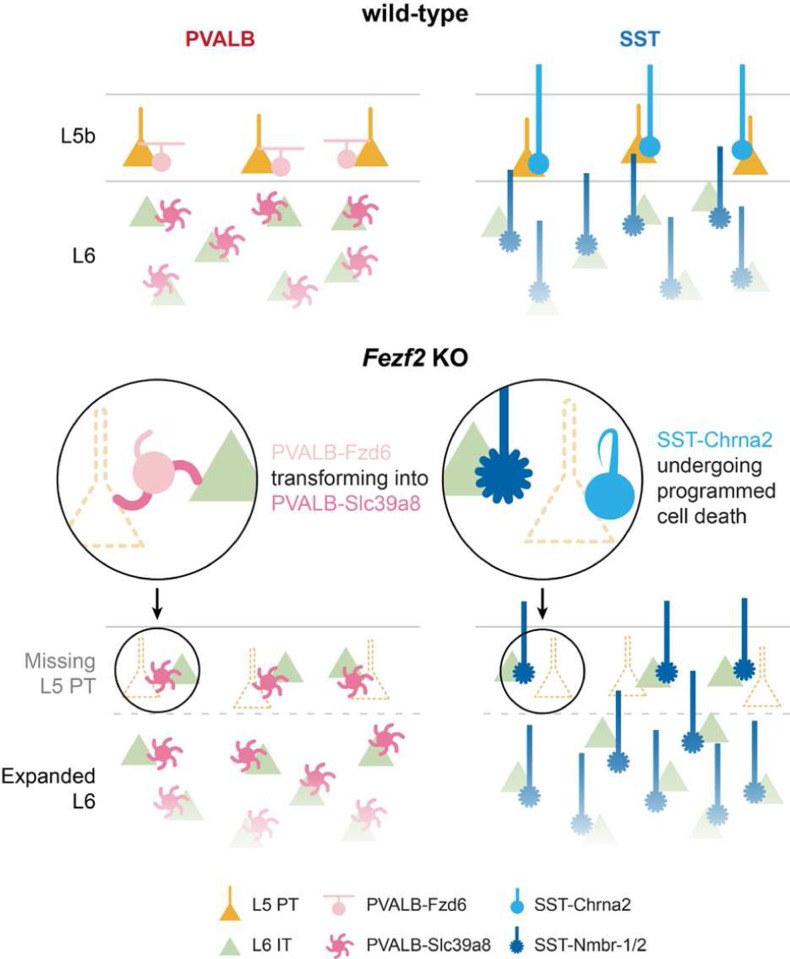
Schematic illustration of how PVALB and SST interneurons adapt to the changes in pyramidal neurons in *Fezf2* mutants.

Taken together, these data suggest that the loss of L5b and gain of L6 PNs in *Fezf2* mutants induce corresponding changes in interneuron subtypes through two distinct mechanisms (Figure 5). While SST interneuron subtypes change their relative numbers through selective apoptosis, PVALB interneurons appear to be able to transform from one subtype to another in response to the changes in pyramidal cell identity in *Fezf2* mutants.

## Discussion

Previous work from our laboratory^4^ and others^5–9^ have revealed a close association between specific PN and interneuron types, forming stereotyped cortical microcircuits that are shared across various cortical regions. It is imperative to understand how this precise circuitry is established during development. Based on previous studies and our analysis, we believe that interneurons enter the cortex already seeded to become specific subtypes, as dictated by their intrinsic gene regulatory networks (GRNs). Upon entering the cortex, these interneurons interact with local pyramidal neurons, receiving external signals that modulate their maturation and adjust the relative abundance of different interneuron subtypes. Importantly, such interactions are subtype-specific, ensuring an inhibitory network that aligns with the composition of local pyramidal neuron types. While the mechanisms for adjusting the subtype proportion of PVALB and SST intemeurons appear different, we believe that they reflect the same underlying principles influenced by their distinct maturation rates. Although PVALB and SST interneurons are born around the same time, SST interneurons rapidly establish their adult identities and begin functioning shortly after arriving in the cortex, while PVALB interneurons mature much later^36–39^. Due to these intrinsic differences in their developmental timetable, the GRNs that define SST subtype identity become relatively fixed as they integrate with PNs, while those for PVALB interneurons remain plastic during early postnatal stages. As a result, in the *Fezf2* mutants, most SST-Chrna2 fail to mature properly in the absence of their synaptic PN partners and undergo programmed cell death. In contrast, the GRNs for PVALB subtypes remain sufficiently flexible to allow the transformation of their identity to the altered balance of PNs in *Fezf2* mutants. However, the few SST-Chrna2 cells that survive tend to shift into L6 and acquire genetic features, accordingly, suggesting that the GRNs of SST interneurons are not entirely rigid. Thus, PVALB and SST interneurons exemplify two extremes in how their intrinsic GRN programs are influenced by extrinsic signals that establish their identities and abundance. This dynamic interplay highlights the nuanced mechanisms that selectively guide interneuron development and their integration into cortical circuits.

## Methods

### Mice

All experimental procedures were approved by the Harvard Medical School Institutional Care and Use Committee and were performed in compliance with the Guide for Animal Care and Use of Laboratory Animals. Mice were housed in a temperature-controlled and humidity-controlled facility and were maintained on a 12–12 h dark-light cycle. All experiments were performed on animals of both sexes. Whenever possible, mice of both sexes are used in experiments. Experiments were not blinded because mice and treatments were easily identifiable as experiments were performed. Sample sizes were not predetermined.

Mouse lines were used in this study include *Fezf2* KO (Ref ^42^), *Rorb*^*Cre*^ mice (JAX #023536), *Tcerg1l*^*CreER*^ mice (JAX #034000), *Pvalb*^*FlpO*^ (JAX #022730), *Rosa26*^*FSF-LSL-tdTomato*^ (*Ai65*) (JAX #021875), *Dlx5/6-Cre* (JAX #008199), *Sst*^*Cre*^ (JAX #018973), *Sst*^*FlpO*^ (JAX #031629), *Chrna2-Cre* (Ref ^19^), *Pdyn*^*Cre*^ (JAX #027958), *Npy*^*flpO*^ (JAX #030211), *Rosa26*^*LSL-tdTomato*^ (*Ai14*) (JAX #007914), *Crhr2*^*Cre*^ (JAX #033728), *Hpse*^*Cre*^ (JAX #037334), *Nkx2.1-Cre* (JAX #008661), *Bax*^*fl*^ (MGI ID:3589203), *Rosa26*^*LSL-h2b-GFP*^ (JAX #036761).

### snRNA-seq

#### Nuclei suspension preparation and library construction.

Brain samples were harvested from mice with specific genotypes and ages, and the cortex was carefully dissected in ice-cold Homogenization Buffer (HB) comprising 0.25 M sucrose, 25 mM KCl, 5 mM MgCl_2_, 20 mM Tricine-KOH, 1 mM DTT, 0.15 mM spermine, and 0.5 mM spermidine. The dissected brain tissue was then transferred to a 2 ml Dounce tissue grinder filled with HB supplemented with 0.15–0.25% IGEPAL-CA630 and 0.2 U/μl RNasin. Tissue homogenization was performed with 8–10 strokes of pestle A followed by 9–10 strokes of pestle B. The homogenate was filtered through a 30 μm filter into a 15 ml conical tube and centrifuged at 500 g for 5 min. at 4 °C using a swinging-bucket centrifuge. The resulting pellet was resuspended in 1X PBS containing 1% BSA + 0.2 U/μl RNasin and then passed through a 40 μm filter. DRAQ5 (BioLegend) was added to the nuclei suspension for sorting of GFP+ nuclei on a Sony MA900 cell sorter using a 70 μm nozzle (see Supplemental Methods). Nuclei were collected in a pre-chilled 0.2 ml PCR tube and counted using a hemocytometer (INCYTO C-Chip). snRNA-seq libraries were prepared using the Chromium Single Cell 3’ Kit v3.1 (10x Genomics), following the manufacturer’s protocol. Pooled libraries were sequenced on NovaSeq 6000 instruments (Illumina).

#### snRNA-seq data processing.

CellRanger (v7.0.0, 10x Genomics) was used with default parameters to map snRNA-seq data to the mouse reference genome (mm 10) provided by 10x Genomics. The gene expression matrices output from CellRanger were then imported into Python v3.9.13 as AnnData objects (anndata v0.8.0). Scanpy v1.9.1 was used as the basic framework for snRNAseq processing. We used Scrublet v.0.2.3^43^ to calculate the potential doublet score for each cell. Unless specified elsewhere, genes expressed in fewer than 3 cells were filtered. Cells were filtered based on the following criteria: n_counts < 15,000, 200 < n_genes < 4,000, ratio_of_mitochondria_genes < 5% (gene symbols beginning with ‘mt-’), ratio_of_ribosome_genes < 5% (gene symbols beginning with ‘Rpl’ or ‘Rps’) and scrublet_score<0.25. After quality control, the raw counts were normalized using the pp.normalize_total function (counts_per_cell_after = 10,000); the normalized counts were log-transformed using the pp.log1p function. The Pearson residuals were calculated from the raw counts for selecting the top 5,000 highly variable genes using experimental.pp.highly_variable_genes function (n_top_genes=5,000). The StandardScaler function (with_mean=False) of scikit-learn v.0.24.0 was then used to scale the Pearson residuals of highly variable genes, followed by the TruncatedSVD function of scikit-learn v.0.24.0 to calculate the Principal Component Analysis (PCA) embeddings for the cells. The unsupervised graph-based Leiden clustering algorithm, pp.neighbors and tl.leiden functions, was used for the clustering based on the PCA embeddings. To sub-cluster specific clusters, the piaso.tl.leiden_local function from our single-cell analysis toolkit PIASO (https://github.com/genecell/PIASO) was used. This function repeats the steps of highly variable gene selection, PCA embedding and Leiden clustering on selected cells while maintaining the clustering results for the remaining cells. After clustering, COSG^44^ was used to identify top marker genes for each cluster, which were cross-compared with well-known marker genes from literature for cell type annotation. For visualization of multiple snRNA-seq datasets across different conditions, we employed the piaso.pl.plot_embeddings_split from PIASO to align cell coordinates from different conditions and scale the gene expression or cell metrics for consistency.

#### Source and processing of public scRNA-seq datasets.

The P28 mouse cortex interneuron snRNA-seq dataset (GEO accession number: GSE164570)^17^, P14 control and *Fezf2* KO mouse S1 cortex snRNA-seq datasets (GEO accession number: GSE158096)^30^, and P1 *Fezf2* Het and *Fezf2* KO mouse cortex scRNA-seq datasets (GEO accession number: GSE153164)^41^ were downloaded and processed by re-mapping the FASTQ files using CellRanger (v7.0.0, 10x Genomics) with default parameters as described above. The processed and annotated SMART-Seq V4 scRNA-seq datasets were downloaded from previous publications^18,45^ and re-annotated.

#### Individual cell-based differential transcriptomic analysis across conditions.

To assess transcriptomic differences across different conditions, we developed a novel algorithm named Emergene. This method performs independent of cell clustering, and hence overcomes the biases associated with existing differentially expressed gene (DEG) analysis methods, which can be skewed by cluster size. First, Emergene identifies genes with localized expression patterns by generating two gene expression diffusion maps. One map diffuses gene expression locally to *k*-nearest neighbors (*k*NN), while the other diffuses gene expression randomly to *k* number of cells. The deviation of these two diffusion maps from the original gene expression map is measured by cosine similarity, a technique employed in COSG. Genes showing specific expression patterns are identified based on the differences between the deviations of the two diffusion maps from the original map. Next, Emergene uses a similar strategy to identify genes with differential expression patterns across conditions. It compares and contrasts the diffusion gene expression pattern to *k*-nearest neighbor on shared cell embeddings across conditions. Together, Emergene identifies a combinatorial gene set that includes genes exhibiting distinctive expression patterns among local cell neighbors under different conditions. Based on the expression level of these genes, Emergene incorporated the permutation test, as used in scDRS for the GWAS risk genes enrichment analysis with improved computational efficiency and scalability, to identify cells with enriched expression of genes that showed specific expression patterns within and across conditions. To determine statistical significance for each individual cell, Emergene generated 10,000 sets of Monte Carlo samples of control gene sets with matched gene set size, expression mean, and expression variance. The *P* values of individual cells were then computed based on the empirical distribution of the normalized expression scores across all cells and all control gene sets. Finally, *P* value for each cell type can be assigned through a gliding threshold (50^th^ percentile by default) based on *P* values of individual cells within each cluster.

#### Feature gene set identification and calculation of expression score.

The identity (ID) feature gene list of a specific interneuron subtype was comprised of the top 200 marker genes identified by COSG that were differentially expressed between the two PVALB or SST subtypes. The immature feature genes were the top 200 differentially expressed (DE) genes identified by COSG that expressed higher at P2 than at P7 for specific interneuron subtypes. DE genes between control and *Fezf2* KO conditions were the top 200 DE genes identified by COSG across conditions for specific interneuron subtypes. The tl.score_genes function from Scanpy v1.9.1 was used to compute the enrichment score of feature gene expression for each individual cell.

#### Triangular Affinity Map for comparative visualization of gene expression similarity.

To generate a visual representation of transcriptomic similarity between one target cell type and three anchor cell types, we employed the Triangular Affinity Map (TAMap) based on gene set expression scores. We first applied min-max normalization to adjust the mean gene set expression scores across various cell type comparisons to a uniform scale, ensuring consistency in the similarity metrics. We subsequently calculated the sizes of three internal angles, each spanned by the target cell type and one of the anchor cell types. These angles represent the similarities between two anchor cell types—such that their sum equaled 360 degrees. Additionally, we computed the edge lengths connecting the target cell type with each anchor cell type based on the similarities between the target cell type and each anchor cell type. For a 2D visualization in a Cartesian coordinate system, the target cell type was positioned at the origin, and one anchor cell type along the positive y-axis. Matplotlib v3.5.2 was utilized to generate the final visual representation.

#### RNA velocity.

To predict how the gene expression pattern of each cell type will change developmentally, we employed scVelo v0.2.4^35^ to infer the RNA velocity from the ratio of spliced and unspliced reads. STAR 2.7.10a was used to quantify the counts of spliced and unspliced reads in individual cells from the P7 *Fezf2* HET and P7 *Fezf2* KO snRNA-seq FASTQ files. Next, we mapped the information of annotated cell types and UMAP coordinates based on cell barcodes. RNA velocities were computed using the scvelo.tl.recover_dynamics, scvelo.tl.velocity(mode=‘dynamical’) and scvelo.tl.velocity_graph() functions, and visualized based on UMAP coordinates with the scvelo.pl.velocity_embedding() function.

### MERFISH

#### Selection of probe set.

To design the MERFISH gene panel that captures the transcriptomic heterogeneity across cell types, developmental stages, and genotypes, we utilized multiple snRNA-seq datasets collected across a wide age range. Top marker genes identified by COSG were combined with known marker genes from literature to generate an initial gene panel for late postnatal ages. Additional genes were added and tested on scRNA-seq collected from younger ages to ensure comprehensive cell type identification across ages. RNA targets were selected based on maximizing unique probe sites per gene for high detection rate while minimizing combinatorial optical crowding for MERFISH imaging. A fluorescently labeled oligonucleotide library probing for 293 combinatorial genes and 4 sequential genes, including GFP was selected. The resulting readout and encoding probes were manufactured by Vizgen Inc. The full list of probes is available in Supplemental Table 1.

#### MERFISH sample processing.

Whole intact brain samples were collected in RNase-sterile conditions, flash-frozen in liquid nitrogen, and moved to 5 mL Eppendorf tubes, and stored at −80 °C for tissue microarray (TMA) construction and MERFISH. First, frozen cortical tissues were collected using 2 mm or 3 mm disposable biopsy punch needles from specific brain regions. The tissue punches were then trimmed uniformly with a sterile razor blade, oriented laterally, and embedded within a pre-formed scaffold of Optimal Cutting Temperature media. On average, six sample punches were assembled into each TMA. All samples were prepared in RNase-sterile conditions for MERFISH imaging according to the procedure described in a previous publication^46^ and using select additional kits and instruments offered through Vizgen Inc. Briefly, the TMA samples were cryosectioned at 10 μm using a cryostat (Leica) at −20°C, and mounted and melted onto fluorescent microsphere-coated, functionalized coverslips, fixed with 4% PFA in 1X PBS, and permeabilized overnight in 70% ethanol. TMA sections were stained using the Cell Boundary Stain Kit (Vizgen, PN 10400009). Following a 1-hour room temperature blocking step in Cell Boundary Block Buffer containing 40 U/μl murine RNase inhibitor, samples were incubated in the primary and secondary antibody cocktails for 1 hour each, with interspersed 1X PBS washes. Primary antibodies against specific proprietary cell membrane proteins and oligo-conjugated secondary antibodies were diluted in Cell Boundary Block Buffer containing 40 U/μl murine RNase inhibitor, with dilution factors of 1:100 and 3:100, respectively. Antibody labeling was fixed again with 4% PFA for 15 minutes. TMA sections were then hybridized with 70 μL of 297-gene probe library solution for 36–48 hrs in a humidified 37 °C incubator with a 2×2 cm square of Parafilm layered onto the surface to prevent evaporation. Samples were embedded in a polyacrylamide gel by incubating the samples in freshly prepared polyacrylamide gel solution (40% 19:1 acrylamide/bis-acrylamide solution, 5M NaCl, 1M Tris pH=8, and nuclease-free water in a dilution of 1:3:3:39; gel solution combined with catalysts, 10% w/v ammonium persulfate solution and NNN’Tetramethyl-ethylindiamin in a dilution of 2000:10:1). To achieve this, the coverslip containing TMA samples were inverted onto the polyacrylamide solution aliquoted on the surface of a Gel-Slick treated, 2×3 inch microslide. Non-targeted molecules were cleared from the gel-embedded sample within a detergent mixture (20X saline-sodium citrate, 10% sodium dodecyl sulfate solution, 25% Triton-X 100 solution, and nuclease-free water in a 5:10:1:34 ratio) supplemented with 0.8 U/^l Proteinase K (NEB P8107S) for 48–72 hours in a humidified 37 °C incubator.

#### MERFISH imaging and post-imaging processing.

Samples were further stained with DAPI and PolyT Staining Reagent (Vizgen, PN 20300021), and imaged using MERSCOPE (Vizgen) with MERSCOPE 300-Gene Imaging Cartridges (Vizgen, PN 20300017). Illumination intensities and exposure times were kept the same in every dataset, capturing images of the whole TMA with both a 10× NA 0.25 and 60× NA 1.4 objective, at 7 z-positions per x–y location, separated by 1.5 μm. MERSCOPE post-imaging analysis used MERlin^47^ to decode positions and copy numbers of target RNA species into count matrices, and Cellpose^48^ to generate cell boundary masks (software version 233.230615.567, segmentation parameter: cell boundary 1).

#### Cell type identification for MERFISH data.

After quality control, four major types of cells were identified based on marker gene expression into excitatory neurons, two groups of interneurons (one group consisting of PVALB and SST, and another consisting of VIP, LAMP5, and SNCG interneurons) and non-neuronal cells. Each group was separately annotated using the marker Gene-guided Dimensionality Reduction (GDR) algorithm developed by our team. GDR integrates annotated snRNA-seq data and unannotated MERFISH data, by first applying unsupervised Leiden clustering to MERFISH data, followed by identification of the top 15 marker genes for both the MERFISH clusters and the annotated snRNA-seq cell types using COSG^44^. GDR calculates expression scores for these marker gene sets across both data modalities, projecting cells into a shared low-dimensional gene expression space that reflects biological identity in each dimension. In this space, cells with similar identities or gene expression patterns are located closely together. To further address technical effects from different modalities, we used the pp.harmony_integrate function from Scanpy v1.9.1 with default parameters to run the Harmony^49^ integration procedure. Following this, Support Vector Machine (SVM) with the radial basis function kernel was used to predict the cell types of cells in the MERFISH data based on the cell embeddings from both MERFISH and snRNA-seq data. SVM was implemented through the svm.SVC function from scikit-learn v.0.24.0, set to kernel=`rbf`, and class_weight=`balanced`. The GDR code is available in the PIASO GitHub repository (https://github.com/genecell/PIASO), and implemented via the piaso.tl.predictCellTypeByGDR function.

#### Analysis of Allen MERFISH data.

The MERISH spatial transcriptomics data of a single adult mouse brain (Allen MERFISH data) with a 500 gene panel was published in Yao *et al.*, 2023^18^. The processed and spatially aligned data with raw counts was downloaded from Allen Brain Cell Atlas (C57BL6J-638850-raw.h5ad; https://alleninstitute.github.io/abc_atlas_access/descriptions/MERFISH-C57BL6J-638850.html). We subset the MOp, MOs, SSp and VISp regions based on the “parcellation_structure” annotation as the data was registered to the Allen CCFv3 (Common Coordinate Framework). The dataset was also further divided into four major cell type groups and annotated separately using snRNA-seq or scRNA-seq reference with GDR as described above. Specifically, 10x Genomics v3 snRNA-seq datasets was used as reference for interneurons. SMART-Seq v4 scRNA-seq datasets were used as references for excitatory neuron and non-neuronal cells. QC and cell type annotation of these reference datasets were performed as described above.

### Slide-seq

#### Library generation and sequencing.

Slide-seq pucks (round, 3 mm in diameter) were generated as described previously^50^. 10 μm-thick coronal sections were obtained from flash-frozen brain samples using cryostat (Leica) and used for generating Slide-seq library immediately, following published Slide-seqV2 protocol^40,51^. Libraries were pooled and sequenced on NovaSeq 6000 flow cells (Illumina). One published Slide-seq data on mouse cortex SSp region was included in this study, puck 200306_02, can be downloaded at https://singlecell.broadinstitute.org/single_cell/study/SCP815/highly-sensitive-spatial-transcriptomics-at-near-cellular-resolution-with-slide-seqv2.

#### Slide-seq data pre-processing.

The sequenced reads were aligned to GRCm39.103 reference and processed using the Slide-seq tools pipeline (https://github.com/MacoskoLab/slideseq-tools; v.0.2) to generate the gene count matrix and match the bead barcode between array and sequenced reads. The spatial barcode recovery step was further optimized with customized algorithm.

#### Cell type mapping using RCTD.

We used RCTD^52^ (now available in the R package spacexr v2.2.1) to map cell types in Slide-seq data based on reference scRNA-seq data. The reference dataset was processed by first retaining only the genes detected in both the Slide-seq data and the reference scRNA-seq data. We then used the R version of COSG (v0.9.0) with the following parameters: mu=100, remove_lowly_expressed=TRUE, expressed_pct=0.1, to select the union set of the top 100 marker genes for each cell type in the annotated scRNA-seq reference. This processed scRNA-seq dataset was then used as input for RCTD. The extraction of distinctive features for each cell type in the reference dataset increased the accuracy of cell type decomposition in Slide-seq data. From the RCTD output, we retained beads classified as ` singlet` or ` doublet_certain` and excluded the rest.

#### Inference of interneuron laminar locations in MERFISH and Slide-seq data.

The resident cortical layer for each interneuron was assigned based on the identity of its five nearest excitatory neuron neighbors. Specifically, the KDTree function from scikit-learn v.0.24.0 with leaf_size=6 and k=5 was used for this purpose.

### RNAscope *in situ* hybridization

For fixed-frozen brain tissue, mice were deeply anesthetized with sodium pentobarbital (Euthasol) via intraperitoneal injection and transcardially perfused with 1X PBS followed by 4% paraformaldehyde (PFA). Brains were then dissected and post-fixed in 4% PFA overnight at 4°C. PFA-fixed brain samples were cryopreserved in 30% (w/v) sucrose and sectioned into 20 μm coronal slices using a sliding microtome (Leica). Brain slices were preserved in the Storage Buffer, comprising 28% (w/v) sucrose, 30% (v/v) ethylene glycol in 0.1 M sodium phosphate buffer, at −80 °C until further processing. For fresh-frozen brain tissue, flash-frozen brain samples were sectioned on a cryostat (Leica) into 19–20 μm coronal slices. mRNA transcripts were detected using the RNAscope Multiplex Fluorescent V2 Assay Kit (ACDBio, 323100), following manufacturer’s protocol for either tissue type with the following modification. Tissue was digested using Protease III for 30 min. at room temperature. The RNAscope catalogue probes used included *Lhx6* (#422791), *Gad2* (#439371), *Sst* (#404631), *Pvalb* (#421931), *Crhr2* (#413201), *Nmbr* (#406461), *Calb2* (#313641), *Hpse* (#412251).

### Image acquisition

Images of RNAscope *in situ* hybridization experiments were collected using a tiling scope (Zeiss Axio Imager A1) with a 10X objective. Images of transgenic mouse line labeling were either collected using a whole slide scanning microscope with a 10X objective (Olympus VS120 slide scanners), or acquired with an upright confocal microscope (Zeiss LSM 800) with a 10X objective (Plan-Apochromat 10x/0.45 M27) or a 20X objective (Plan-Apochromat 20x/0.8 M27) to better appreciate the cellular morphology. Images of viral genetic labeling of SST-Hpse and SST-Crhr2 were imaged using another confocal microscope (Leica Stellaris) with a 10X objective (HC PL APO 10x/0.40 DRY). Stitching of image tiles was mostly performed using acquisition software, except for some used Stitching plugin in FiJi^53^.

### Statistics

Most of the statistical tests in this study employed two-sided non-parametric tests due to the small sample sizes (n<25) and the non-normal distribution of single-cell expression data. Boxplots display the median, the 25^th^ percentile, the 75^th^ percentile, with two whiskers that extend to 1.5 times the interquartile range. A summary of statistical test results is provided in Supplementary Table 2.

## Extended Data

## Data Availability

The data have been deposited at the Gene Expression Omnibus (GEO) under accession number GSE272706, and at the Single Cell Portal: https://singlecell.broadinstitute.org/single_cell/study/SCP2716/pyramidal-neurons-control-the-number-and-distribution-of-cortical-interneuron-subtypes.
